# Tollerability of incisional hernia repair in local anesthesia for old patients in a day surgery setting

**DOI:** 10.1186/1471-2482-13-S1-A18

**Published:** 2013-09-16

**Authors:** Angelita Giglio, Giovanna Brancato, Maurizio Ristagno, Antonio Biondi, Marcello Donati

**Affiliations:** 1 Department of Surgical Sciences, Organ Transplants and New Technologies.General Surgery and Week Hospital Unit. University Hospital of Catania, Via S.Sofia 78. 95123, Catania, Italy; 2 Department of Surgery, General and Oncologic Surgery Unit, Vittorio-Emanuele University Hospital of Catania. Via Plebiscito 628. 95122, Catania, Italy

## Background

Nowadays the gold standard of the incisional hernia surgical treatment seems to be the prosthetic repair. In the last 15 years many Authors published about the advantages of the laparoscopic treatment in front of the Open one, even if this issue till today remains controversial[1]. Anyway our group from 18 years is engaged on a clinical application of a third way of incisional hernias repair with an open prosthetic technique under local anesthesia which feasability was already demonstrated[2]. Other published experiences already exists[2]. At the beginninig of our experience we started with small incisional hernias[3]; over the time we submitted to procedure even incisional hernias with orifice’s diameter till 10 cm. Intraoperative sufferance of patients is questioned. The aim of this work is to show the patient’s tolerance to this open preperitoneal mesh technique of incisional hernia repair in local anesthesia in the elderly.

## Methods

Between January 1994 and December 2011, 164 patients underwent an open mesh surgical procedure for incisional hernias. In 103 the operation was conducted under local anesthesia. They were divided in two sub-cohorts of patients following age criteria as Group A<65 yrs: 64 pts.(62.1%) and Group B >65 yrs:39 pts( 38.9%). All patients were submitted to local anesthesia procedure after overcoming 3 selection criteria: reducibility of hernia sack, absence bowel obstructive symptoms related symptoms, maximum diameter of hernia’s orifice till 10 cm[4]. detected during preoperative work-up through US[5] and CT scan. The collective with consistent co-morbidity (51/103, 49.5%), respectively gr. A 25 pts (39%) and gr. B 26 pts. (66.6%).

We compared both Groups for referred intraoperative pain (reported by VAS score ranging from 0-10), use of intraoperative sedation or other drugs, cardial alterations during procedure, time of postoperative feeding and deambulation, time of discharge and long terms results for recurrences.

## Results

We observed only 3 conversion to general anesthesia (~3%) 2 in gr.A and 1 in gr.B, mainly due to psycological stress (they all referred a VAS ranging between 2 and 4). All the other 100 patients completed the procedure under local anesthesia (~97%). The average operative time was in gr. A 102 min. vs. gr.B 110 min, sedation was used in gr.A n 10 pts(17.4%) vs in gr.B in 7 pts. (18.3%). Never a drainage was positioned in both groups. Intraop. pain (light pain VAS:0-3) was referred in gr. A by 8 pts. (12.9%) and in gr. B by 3 pts.(7.8%), no middle (VAS:4-7) and strong pain (VAS:8-10) were observed in both groups. Cardial alterations (bradycardia) were observed respectively on 6 pts. (gr.A:9.6%) and on 5 pts.(gr.B:13.1%). All patients in both groups deambulated immediately after operation and had first oral intake after 2 hours without any difference in both groups.

Sixtyfour of them were discharged within 24 h. (64%) distributed as follows: 40 gr.A (64.5%) vs 24 in gr.B (63.1%). No wound complications of clinical importance were observed in all the collective of patients. In follow-up (range 16-220 months) only 4 recurrences (4.1%) in local anesthesia group were registered: 3 in gr.A and 1 in gr.B.

## Conclusions

Our data clearly are showing that local anesthesia for incisional hernia repair with a open preperitoneal mesh technique is feasible, safe, and effective. The preoperative selection criteria are effective in young so like in old patients. The old patients showed the same intraoperative tollerability as the young ones. A majority of patients following the above mentioned selection criteria can be managed in day surgery even in the elderly. In the old patients considering the comorbidity and consequently the high intraoperative risk, this method should be considered a valid alternative choice, being cost-effective and showing very good long-term results. In our opinion the local anesthesia approach should be better considered by surgical community as a third choice for the surgical management of incional hernia in selected populations of patients, and should find a defined position on the armamentarium of surgical options, expecially regarding old patients.
